# Effect of ZnO/EAF slag doping on removal of methyl red dye (MR) from industrial waste water

**DOI:** 10.1038/s41598-024-77809-5

**Published:** 2024-11-08

**Authors:** D. A. Wissa, Nadia F. Youssef, Christen Tharwat

**Affiliations:** 1 Solid State Physics Department, Physics Research Institute, National Research Centre, Giza, Egypt; 2 Raw Building Materials Technology and Processing Research Institute, Housing and Building National Research Center (HBRC), Giza, Egypt

**Keywords:** EAF slag NPs, ZnO NPs, MR dye, Sunlight, Photocatalytic effect, Materials science, Physics

## Abstract

Zinc oxide doped with EAF slag (ZnO/ EAF slag) nanoparticles in different contents (10, 20) % of waste were synthesized in a controlled and reproducible way using spin-coater. The produced nanomaterial’s physicochemical and structural characteristics were ascertained by means of particle size distribution, TEM, SEM, UV-Vis spectroscopy, XRD, FTIR, and XRF. The role and effect of EAF slag with constant percent doping with ZnO on the ability to remove pollutants was determined by observing the methyl red (MR) elimination in an aqueous solution at λ_max_ = 413 nm and MR dye removal concentration was evaluated from its optical density. Irradiation of the compounds in sunlight intensity 250 KW/nh.m^2^ and temperature 36 °c resulted in a larger degree of MR removal from the solution, resulting in ZnO/EAF slag samples exhibiting increased photo activity. As a conclusion ZnO nanoparticles saturated with 20% EAF slag as a waste material were the most efficient in removing methyl red (MR) achieving ~ 96% removal and a completely transparent solution after 2 h of testing.

## Introduction

Wastewater contaminated with dyes coming from dyes industries (even dyes industries or industries using dyes in their process) become a huge environmental problem, the removal of these dyes might be accomplished by a variety of methods, including chemical degradation and absorption, but search for simple, new, and cost-effective solutions for the total elimination these sorts of pollutants is a difficult challenge^1^. The textile and dyestuff industries already use a lot of these synthetic dyes. The pharmaceutical, cosmetics, paper, and food sectors also make extensive use of them. In particular, dyes have been found to be among the most harmful substances in textile effluents since around 30% of the dye content is still in the aqueous phase^2^. The absorption method is a cheap and simple way to remove pollutants, but it must be combined with other procedures. In heterogeneous photocatalysis, a semiconductor absorbs a photon and creates a pair of electron holes on the surface, which produces highly reactive species that can break down a variety of contaminants present in residual fluids^1,3^. Cu_2_O, TiO_2_, V_2_O_5_, ZrO_2_, Fe_2_O_3_, and ZnO nanoparticles (NPs) have all been explored as promising photocatalytic materials^4–8^. Despite this, the broad straight bandgap, high electronic mobility, lack of toxicity, and ease of morphological control of nanostructured zinc oxide (ZnO) make it a desirable catalyst^9^. ZnO and other metal oxide-based compounds have gained significant interest as adsorbent materials because of their potent adsorption capacity and large surface area^10^. ZnO is recognized for its chemical stability, biocompatibility, non-toxic nature, and cost-effectiveness. Due to these attributes, it has been utilized for various applications, including serving as a monometallic catalyst^10,11^. Another benefit for the practical application of photocatalyst in wastewater treatment is that ZnO is more suited for the photodegradation of dyes in the presence of sunshine^12^.

Lakshi et al.^12^ examined the MG dye’s photodegradation by the synthesized ZnO nanoparticles when exposed to sunlight. ZnO nanoparticles of varying morphologies have been documented to function as a photocatalyst in the UV light-induced degradation of phenol as well^13^.

EAF slag as a waste is used as an alternative to rock fragments contained in concrete, or as lose ground cover material. It can be commonly used as road base material in road paving process. In some states, EAF slag is also marketed for use in landscaping^14^. Slag is a by-product that turns into a dark gray, angular-shaped, rough-textured stone after cooling from temperatures as high as 1300 °C to room temperature. It is sometimes referred to as “oxidizing slag” or “black slag.” EAF slag characteristics are influenced by the kind of steel that is produced in the furnace, the makeup of the scrap, how the slag is cooled, and weathering processes^15^. A comprehensive study utilizing the water sprayed EAF slag from another factory of the same company (EZZ Steel) was conducted and published^16^. Two wastewater samples were collected from this plant’s main combined drainage line at its entrance and discharge. It discussed the applicability of WS-EAF steel slag for removal of Cd and Mn ions from aqueous solutions and industrial wastewaters through the adsorption process. The findings demonstrated that temperature, contact time, concentration, adsorbent dosage, particle size, and solution pH all affected the removal of Cd (II) and Mn (II) by WS-EAF steel slag^16^. As far as we are aware, there are no researches issues exist about the use of ZnO/ EAF slag doping to remove Methyl Red dye (MR) from industrial effluent.

The EAF slag was used to purify industrial waste water in a previous work, but in our current work it was mixed with zinc oxide and used for a specific dye removal which was not tried by other researchers before. So the current study aims to utilize waste to remove dye from water. This work aims to prepare a mixture of ZnO doped EAF slag waste with different contents suitable for the decomposition of methyl red dye from aqueous solution. The use of EAF slag when mixed with ZnO may be a novelty in this aim of enhancing this process by using inorganic materials such as ZnO doping with an activator such as EAF slag. This is environmentally beneficial for both targets of utilizing scrap waste from one side and removing dyes, purifying recycled water and make it possible to reuse it from the other side.

## Experimental work

### Materials

All of the analytical grade chemicals were used without any purification. Methyl red (MR) dye with M.W. 269.31 and purity 97% is a pH indicator; it turns pink/red at 4.4 pH and lower, yellow at 6.2 pH and higher, and orange in between supplied by s.d.fine CHEM limited, India. Zinc oxide (ZnO) powder with M.W. 81.38 and purity 99% used was purchased from LOBA CHEMIE, India. EAF slag used is a waste from the manufacture of iron produced by the EZZ steel company in Egypt (established 1998). The production depends on the Electric Arc Furnace (EAF) technology to re-melt iron scrap to a temperature up to 1800 °C. It approves the Batch Melting Process, where the batch may be composed of iron metallic source such as iron scrap, and direct reduced iron, and non-metallic materials to help the slag formation and purification of the produced iron. These materials could be CaO and MgO as fusion aids and comes from both lime and dolomite as natural raw materials. The batch contains also charcoal as a source of carbon to help stirring of the molten iron and formation of the foaming slag. Iron oxide Fe₂O₃ is magnetically separated then added to the furnace as scrap batch. It also stated that CaO with other elements oxides found in the scrap is expected to melt at 1800 °C as fumes where it can be separated by filters as EAF Dust, away from the slag .In all cases the added materials to the scrap before melting aim to act as fusion aids and alkalinity adjustment then purification of the product. The slag was used as produced from the company with no intention to change its chemical composition^17^. It is expected that the EAF slag may contain a complex mixture of silicates and oxides. It constitutes about 13–20% of the produced steel. Regarding the big production capacity of 7 million tons per year, the company established a production line that crushes slag and mills it to sell part of the slag to be mixed with cement for non-reinforced concrete. It was laboratory milled to obtain the very fine particles required for Dye removal work.

### ZnO doping EAF slag

ZnO doping EAF slag were prepared using simple mixing method, for ZnO we used two concentrations 0.5gm & 1gm, and the slag concentration with respect to ZnO were 10 & 20 &25 wt%.

### Methyl red dye removal by ZnO and ZnO/EAF slag nanoparticles

**Fig. 1:**
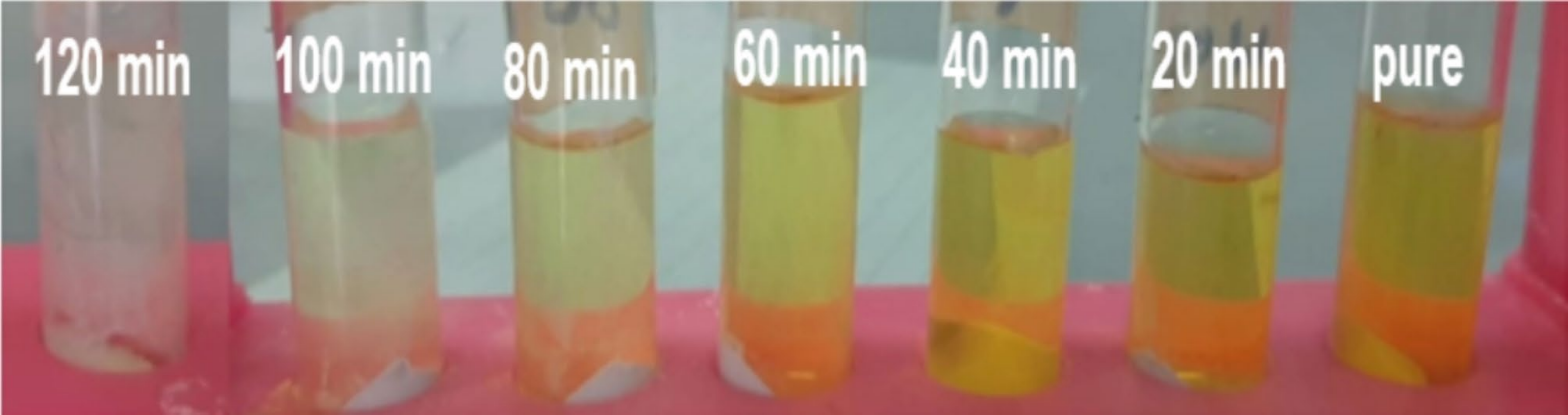
MR dye removal after 2 h.

In a typical stoichiometric case, pure ZnO NPs (0.5 and 1) gm was added to MR dye solution (0.02 gm, 500 mL) and stirred by magnetic stirring. To create the adsorption equilibrium between the photocatalyst and the MR dye, the resultant solution was agitated for two hours under the sunlight. Figure 1 showed periodically the reaction mixture was removed every 20 min by using a syringe tube and then centrifuges (REMI, R-8 C BL Bench Top Centrifuge 5000 rpm for 30 min) to separate the suspended ZnO nanoparticles from the solution. The dye concentration decreases due to reaction between ZnO NPs and MR dye was estimated from its optical density. The above steps are repeated when adding (10, 20 and 25) % of the mixture EAF slag with zinc oxide^18^.

## Assessment of the inorganic raw materials

Fine powders of both inorganic materials used during this work were assessed through 3 examinations as follows^19^.

1. An examination using the X-ray fluorescence method (XRF). Axios (PW4400) WD–XRF Sequential Spectrometer (Panalytical, Netherlands) was used for the chemical analysis, with a radiation tube (Rubidium) Rb kα operating at 50 kV and 50 mA.
2. The powder X–ray diffractometry analysis was carried out using X’Pert Pro PANanalytical –Manufactured by Panalytical B.V. Co., Netherlands. With its computer certified program and the aid of the international center of diffraction data base (ICDD), in order to detect its mineralogical composition.
3. The fineness of particle size and its normal distribution was examined using the Laser Scattering Particle Size Distribution Analyzer device (Horiba LA950), based on volume distribution measurements.
4. Transmission electron microscope (TEM) The particle size of the utilized nanomaterial was measured using TEM model: JEM-HR 2100 and accelerating voltage 200 kV, Japan.
5. The FEI Quanta 250 SEM model is a field emission scanning electron microscope (FESEM) that is used for chemical and structural examination of metallographic specimens as well as dynamic in situ investigation of a variety of samples in their natural state. Magnification of up to one million times and resolution of three nanometers.
6. UV–Vis spectrophotometer (USB 650 Fiber Optical Spectrometer Model No. DDS-3 × 25 USB) was measured the absorbance (A) at appropriate wavelength corresponding to the maximum absorption of Methyl red (i.e., λ_max_ = 413 nm) during the photocatalyst at room temperature. The diffuse reflectance spectrum (DRS) Spectrophotometer [Jasco, V-570] Spectral range from 190 to 2500 nm.
7. ATR-FTIR, Bruker VERTEX 80, Germany combining Platinum Diamond ATR, which uses a diamond disk as an internal reflector in the 4000–400 cm^− 1^range with resolution of 4 cm^− 1^, refractive index of 2.4.
8. PL spectrometer consists of He-Cd laser (532 nm), Sumitomo optical He closed cycle cryostat coupled with THR 1000 mono-chromator (Jobin-Yvon), excitation power source 20mW, and FTIR spectrometer Nicolet 5700. The following spectral range (100–800 nm) can be measured with excellent precision and sensitivity using the PL setup.

## Results and discussion

### Characterization of waste EAF slag and zinc oxide

Table 1 shows the chemical composition of both materials used for the mixtures, as examined by XRF.

**Table 1:** The chemical composition (XRF).

Main Constituents	EAF Slag Wt%	ZnO Wt%
**ZnO**	0.01	98.40
**SiO**_**2**_	14.20	0.04
**TiO**_**2**_	0.70	----
**Al**_**2**_ **O**_**3**_	7.68	0.04
**Fe**_**2**_ **O**_**3**_	37.20	----
**MnO**	4.15	----
**MgO**	1.49	----
**CaO**	31.10	----
**K2O**	0.03	----
**Na**_**2**_ **O**	0.18	----
**P**_**2**_ **O**_**5**_	0.33	----
**SO**_**3**_	0.43	0.02
**Cl-**	0.05	----
**Cr**_**2**_ **O**_**3**_	1.92	----
**PbO**	----	0.39
**WO**_**3**_	----	0.37
**CdO**	----	0.09
**BaO**	0.28	----
**SrO**	0.08	----
**Nb**_**2**_ **O**_**5**_	0.02	----
**L.O.I**	0.01	0.63
**Total**	99.95	99.98

**Fig. 2:**
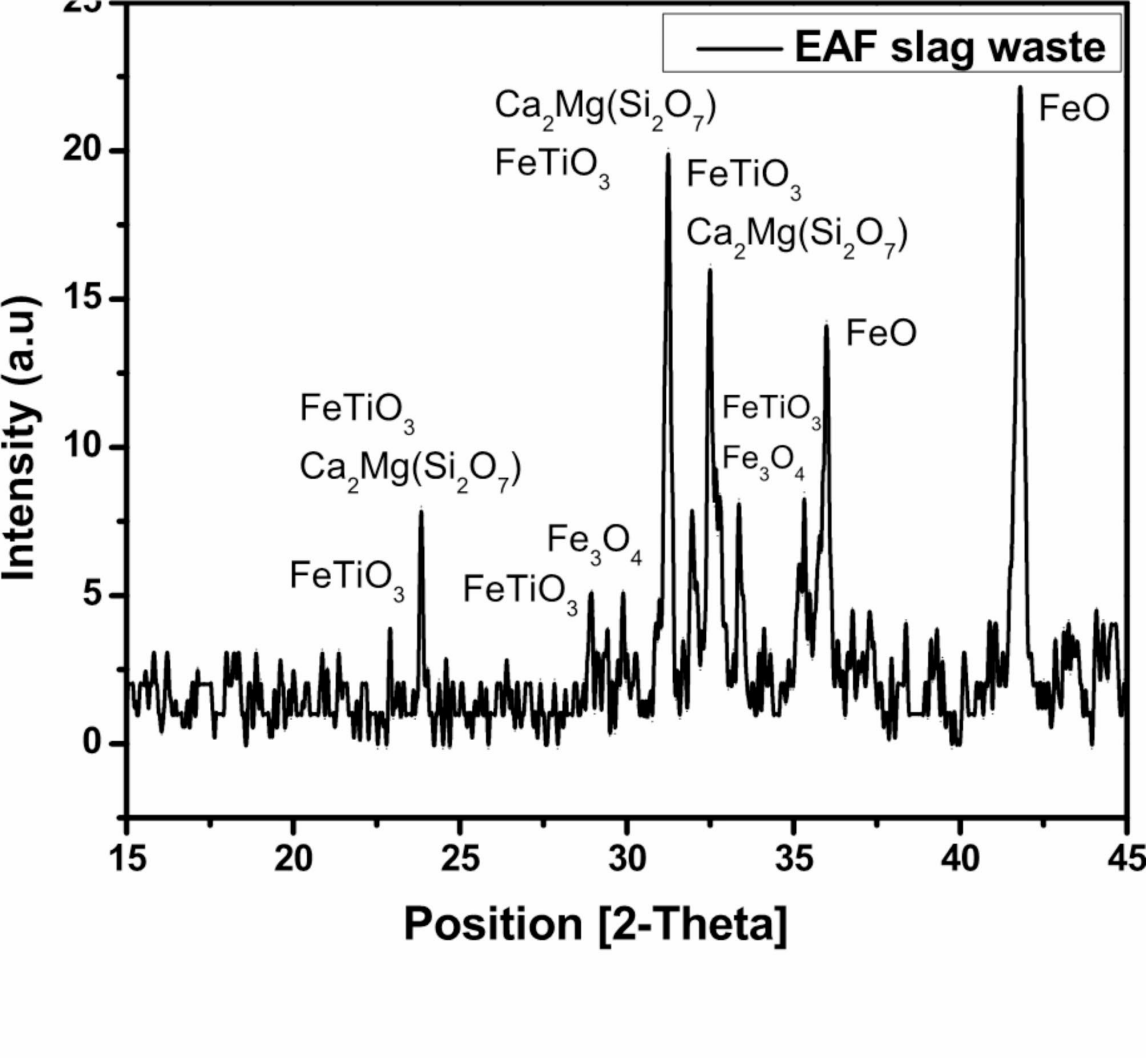
XRD pattern of EAF slag waste.

**Fig. 3:**
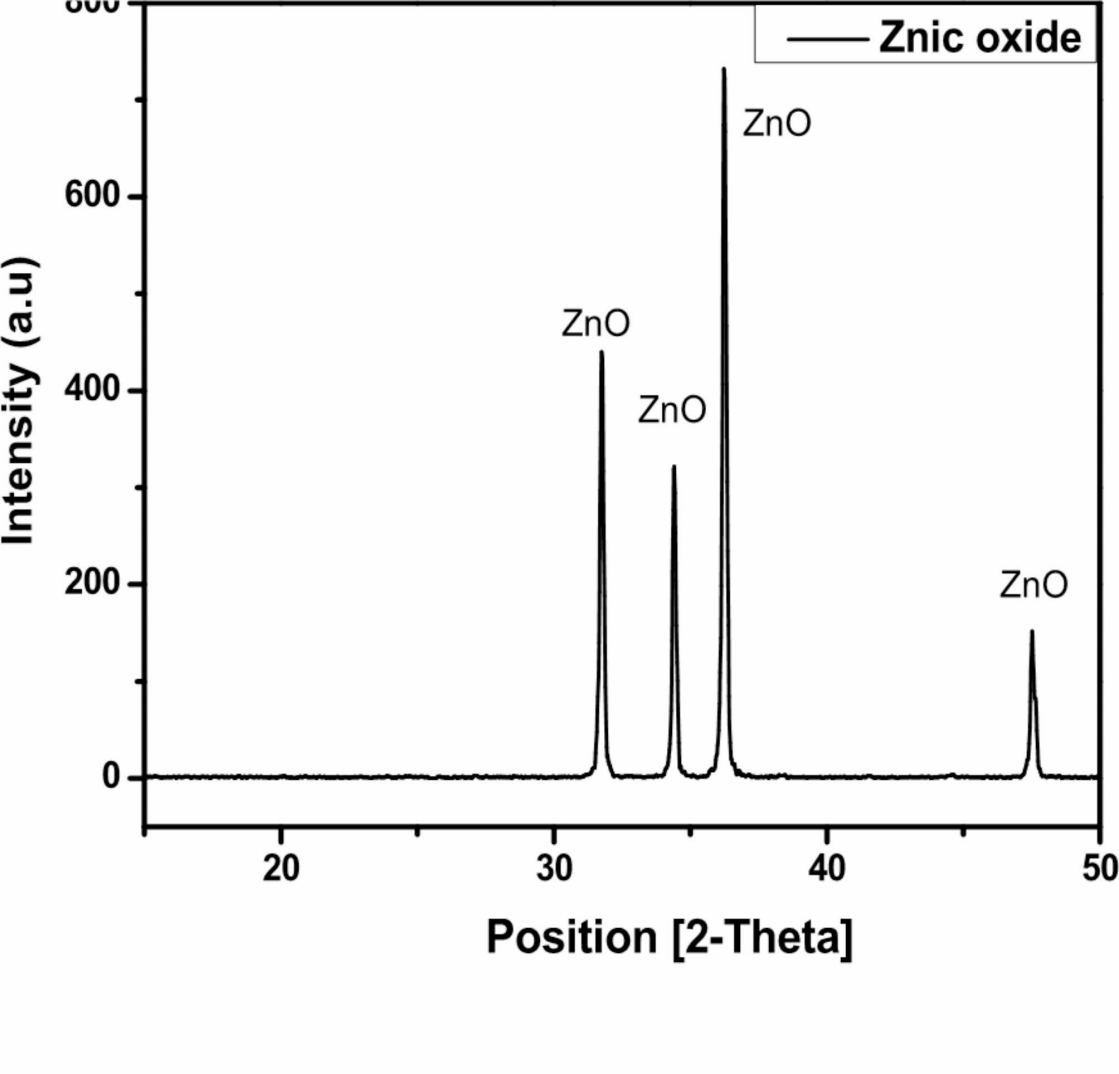
XRD pattern of zinc oxide.

The pattern in Fig. 2 showed that the slag contains several mineral phases. These minerals are: Akermanite ( Ca_2_ Mg ( Si_2_O_7_ )), Wustite, syn (FeO), Ilmenite (Fe Ti O_3_), Magnesite (MgCO_3_), Magnetite( Fe_3_O_4_), Calcium sodium magnesium aluminium iron disilicate (Ca_1.53_ Na_0_._51_ ) ( Mg_0.39_ Al_0.41_ Fe_0.16_ ) Si_2.0_ O_7_). This result agrees with the chemical composition shown in Table 1 where silica, alumin, iron, Calcium and magnesium oxides form the highest weight percentages. Although Manganese oxide was present on chemical analysis, but it did not show up as any mineral phase on XRD analysis while titanium showed up as Ilmenite. This could be sometimes due to technical reasons. Figure 3. illustrates the XRD pattern for ZnO NPs; distinguishing peaks with a Zincite hexagonal structure were shown. Zincite, syn (ZnO) was the only mineral phase that showed up on XRD pattern of Zinc oxide used in this work. Table 1 showed some oxides as Tungsten trioxide (WO_3_) which could be no more than contamination. Also sharp and strong peaks of EAF slag and ZnO NPs indicate to the high crystallinity for the samples^20,21^.

**Fig. 4:**
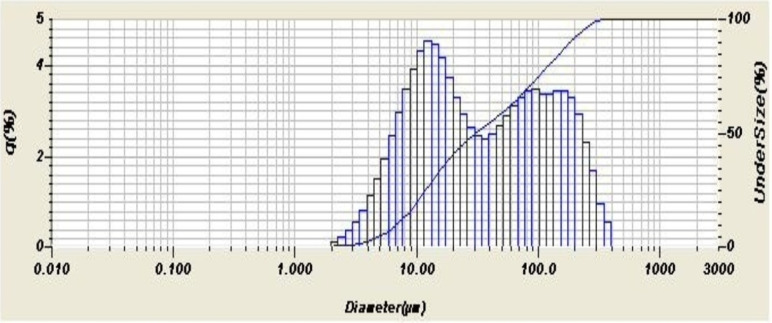
Distribution of Particle size for EAF slag sample.

**Fig. 5:**
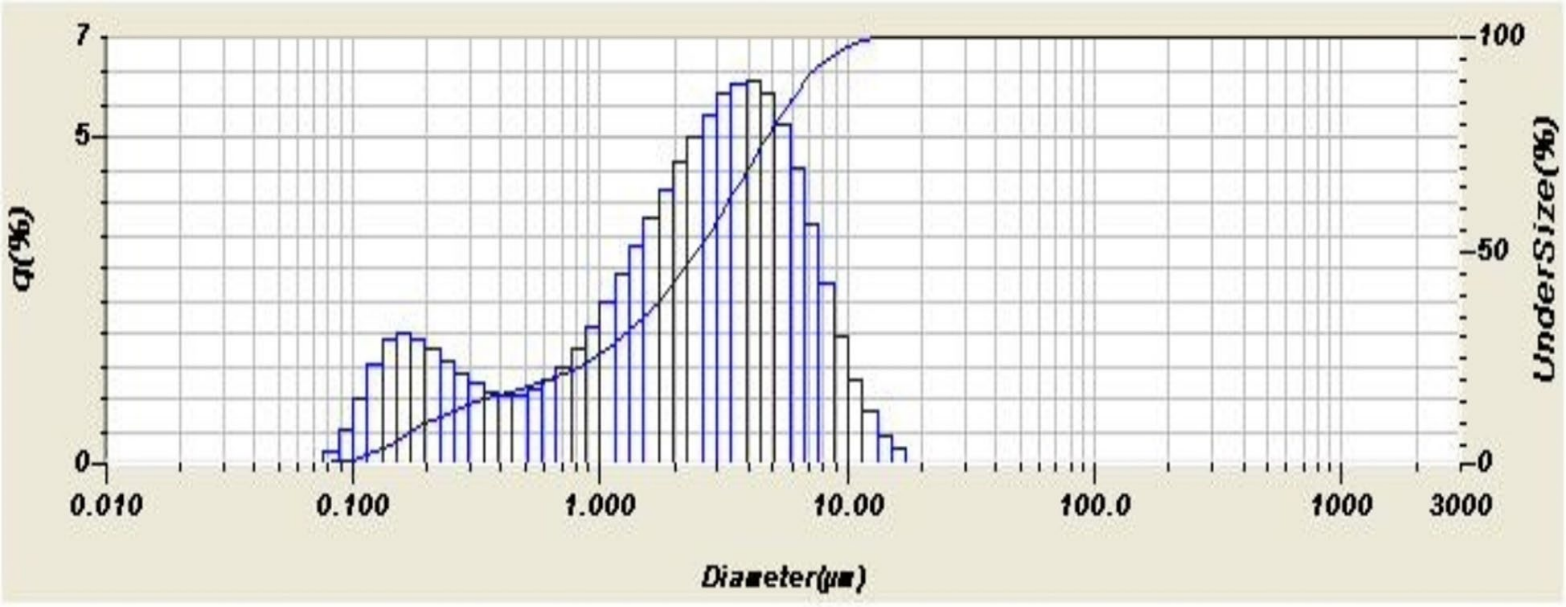
Distribution of particle size for ZnO sample.

Figure 4 shows the particle size distribution of the iron slag waste, while Fig. 5 shows the particle size distribution of zinc oxide. Both patterns show 2 peaks of the normal distribution each. Both powders are fine enough to be in micron ranges even if there is a difference in their finesse. The analyzer’s direct and calculated parameters provide an explanation of the particle size distribution analysis as shown in Table 2. As will be shown later, the fineness of the powder mixtures was an effective factor for the coming results.

**Table 2:** Particle size distribution of the waste and oxide.

Item	EAF slag waste	Zinc oxide
**Mean size**,** µm**	68.03	3.160
**Median size**,** µm**	31.719	2.502
**Mode size**,** µm**	12.400	4.170
**Diameter on cumulative of 10%**,** µm**	7.081	0.217
**Diameter on cumulative of 90%**,** µm**	186.255	8.951
**Size range**,** µm**	1.981to 394.244	0.76 to 17.377

### TEM

**Fig. 6:**
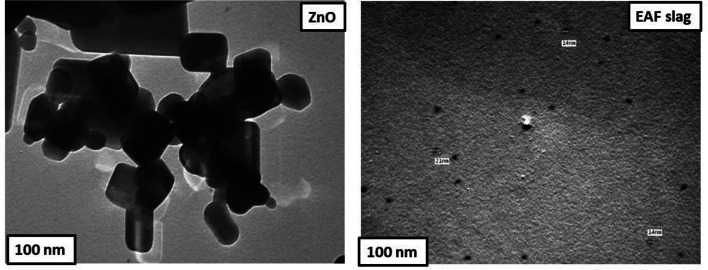
TEM of ZnO and EAF slag nanoparticle.

To get direct evidence about the nanosize and internal shape of the nanoparticles the TEM was used. Figure 6 confirmed the nanostructure for ZnO and EAF slag NPs, the shapes for ZnO and EAF slag NPs are nanorod shaped and spherical like shape respectively. Also particle size for these nanomaterials under nanoscale at 100 nm^22,23^.

### FESEM

**Fig. 7:**
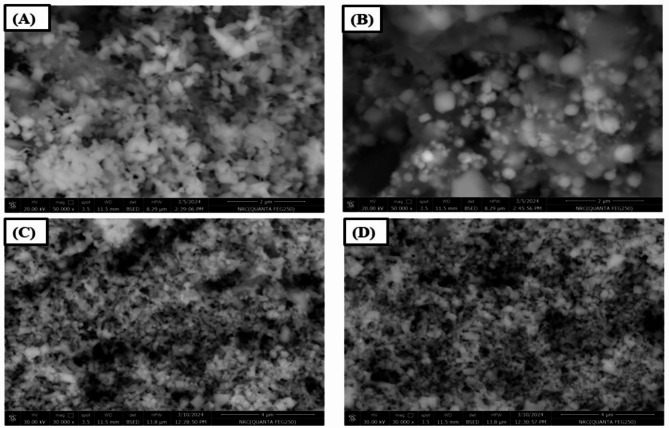
SEM for ZnO, EAF slag and ZnO doped EAF slag nanoparticles different contents.

The surface morphology of the catalyst is one of the important factors affecting on Photocatalytic efficiency. Figure 7. represents surface distribution images for ZnO and EAF slag NPs. The SEM images shows uniform cube like shape of ZnO as shown in Fig. 7A but the image appears aggregated spherical shape related to EAF slag waste as shown in Fig. 7B^24^. From Fig. 7C and D show SEM image of ZnO doping EAF slag at different percent 10 &20% and represents homogenous distribution for the particles due to mixing ZnO and EAF slag nanoparticles.

### UV-Vis optical

**Fig. 8:**
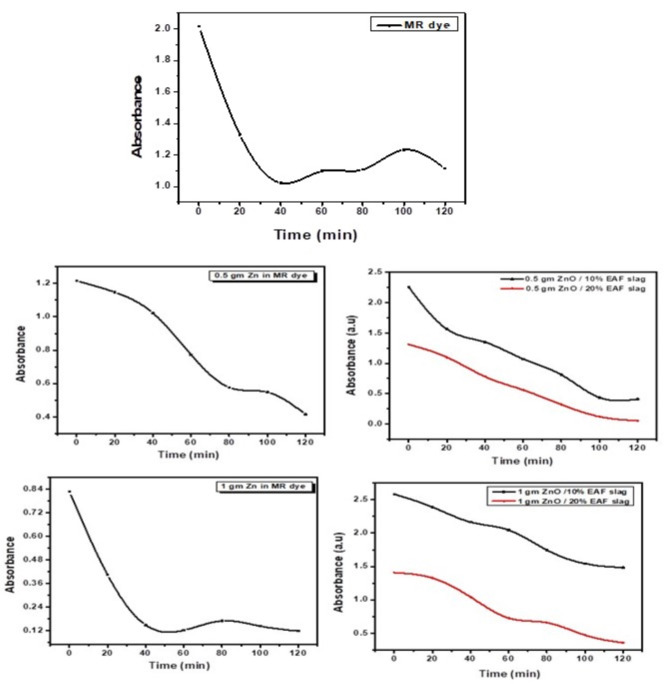
The absorbance spectra of MR dye, ZnO and ZnO doped EAF slag different contents during the Photocatalytic.

The Removal of MR dye by ZnO and ZnO / EAF slag nanoparticles was measured using UV-Vis spectrophotometer. MR dye removal concentration was evaluated from its optical density at λ_max_ = 413 nm, which we found it vary according to PH value^25^. Figure 8, appears the absorbance of MR dye was measured before and after exposure to the sun light at intensity250 KW/nh.m^2^ and temperature 36 °C for different intervals of time, Fig. 8 shows that decrease in MR dye concentration when increasing irradiation time due to photo catalytic activity, this is due to reaction between ZnO and ZnO / EAF slag NPs with MR dye was expressed as a function of time. Figure 8 shows absorption related to time in case of adding dosage from (0.5, 1) gm ZnO only and with adding 10 and 20% EAF slag NPs in 25 ml MR dye at wavelength 413 nm.

### Dye removal efficiency under UV irradiation

**Fig. 9:**
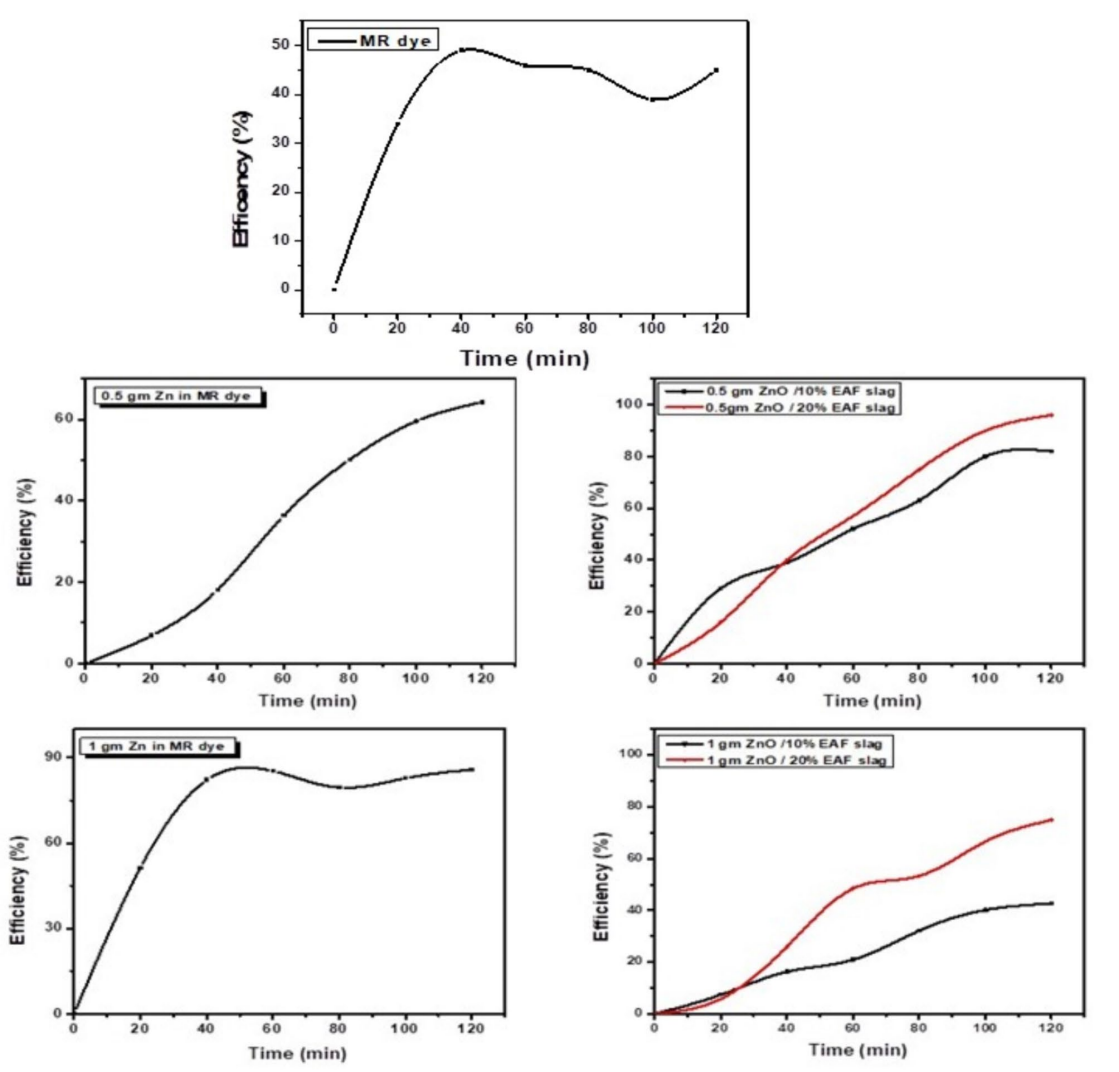
Efficiency of MR dye removal before and after addition ZnO and ZnO doped EAF slag different contents.

Figure 9, show efficiency of MR dye removal before and after addition of different dosage from ZnO and ZnO doped with different contents from EAF slag, Eq. (1) used to calculate the efficiency percent %.

1 $${\rm Efficiency} \% = ({\rm A}_{0}-{\rm A}_{\rm t}) /{\rm A}_{0}\,\times\,100\% .$$

Here A_0_ is initial absorbance of MR dye and A_t_ is absorbance of MR dye at each time “t”.

Figure 9 shows that, the efficiency of MR dye removal before any additions reached under 50% after 2 h.

After addition 0.5 gm ZnO, the efficiency of MR dye removal reached up to 36% in 60 min and 65% at the end of 120 min. The efficiency after addition of 0.5 gm ZnO doped with 10% EAF slag around 52% in first hour, it was later increased to 82% in 120 min was notice. Additionally, with the addition of 0.5 gm ZnO doped with 20% EAF slag, the efficiency increases in the first hour, but this figure shows that dye removal efficiency slows down in the second hour. Moreover, after 120 min, 96% efficiency was attained. Also, in cased of dosage 1gm ZnO the efficiency increased up to 85% in the first hour and after that decreased, this is due to interaction caused between ZnO with MR dye solution. When added 10 wt% EAF slag content doping with ZnO, the efficiency start to increase with small rate reached to 22% in 60 min and 42% at the end of 120 min. After added 20 wt% EAF slag content doping with ZnO, the efficiency of MR dye removal improved and increased to 50% in the first hour and reached to 75% in the second hour. Then we noticed that when 1gm ZnO doped with 25% EAF slag the maximum efficiency we get was 10% removal only and these maybe due to the interaction between ZnO doped EAF slag and MR dye molecules.

### FT-IR spectra

**Fig. 10:**
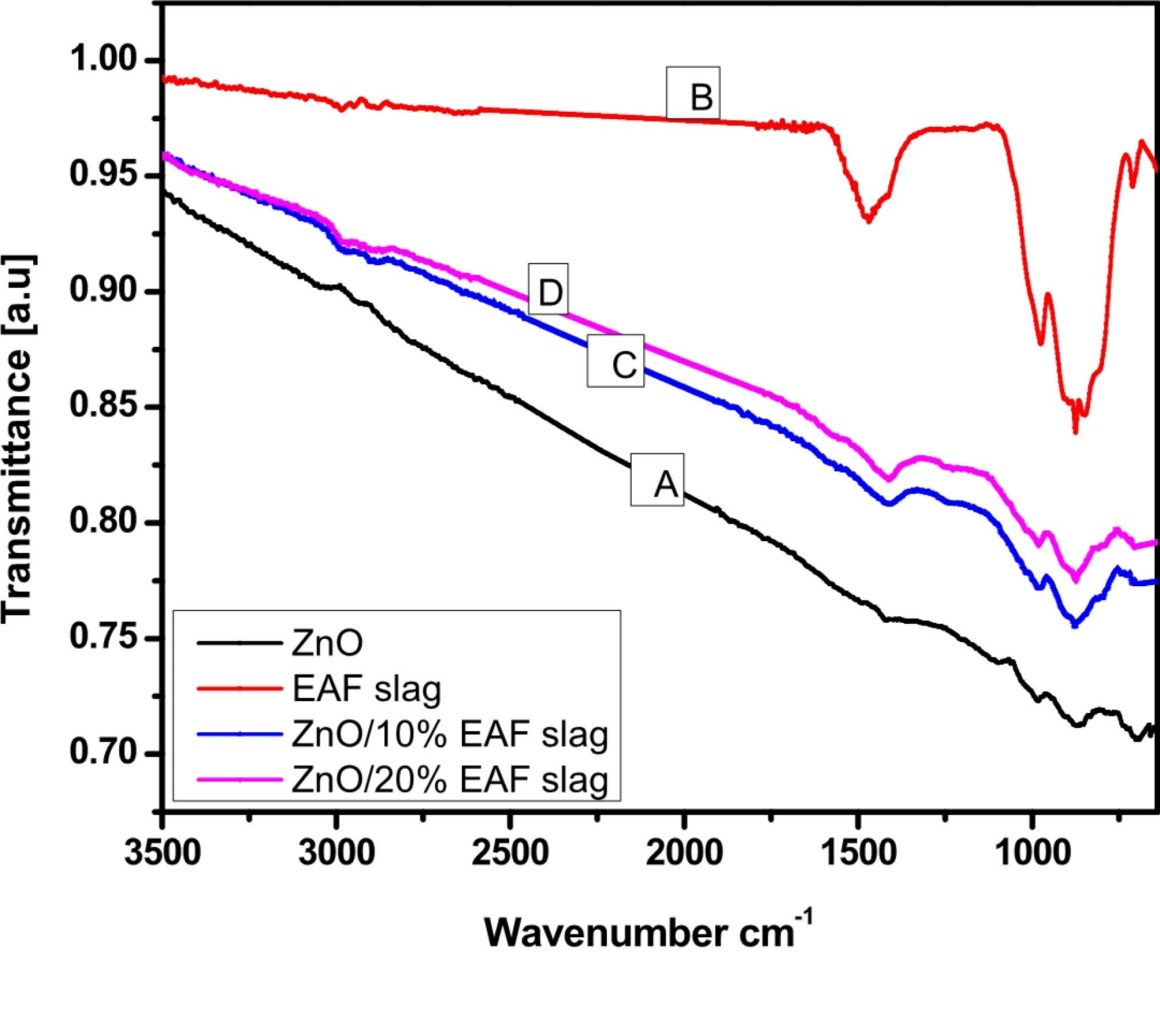
FTIR spectrum of ZnO, EAF slag and ZnO doped EAF slag with different contents.

Figure 10A, clears the FTIR spectra of ZnO NPs, a band at 864 cm^-1^ is observed which is attributed to Zn-O band stretching. Figure 10B, presents the FTIR spectra related to EAF slag, a band is observed at 875 cm^-1^ belongs to the asymmetric stretching vibration of the Al-O bond of the AlO_4_ groups^25,26^, which suggests the presence of gehlenite.While the stretching vibration of the Si-O bond, which originated from calcium silicate, is attributed to the band at 975 cm^− 1^^24^.Additionally, the band at 1510 cm^− 1^ that corresponds to O-C-O stretching vibrations^27–29^. The presence of carbonates and traces of calcite (CaCO_3_) is indicated by this peak. Figure 10C&D, presents FTIR spectra for a mixture of ZnO and EAF slag. From spectrum it’s clear that band intensity decreases and shift to the low wavenumber. Already a band in region from 1121 to 1430 cm^-1^ for a mixture of oxide and only ZnO can be attributed assigned to the asymmetric stretching modes of oxygen present in sample.

### Optical properties

**Fig. 11:**
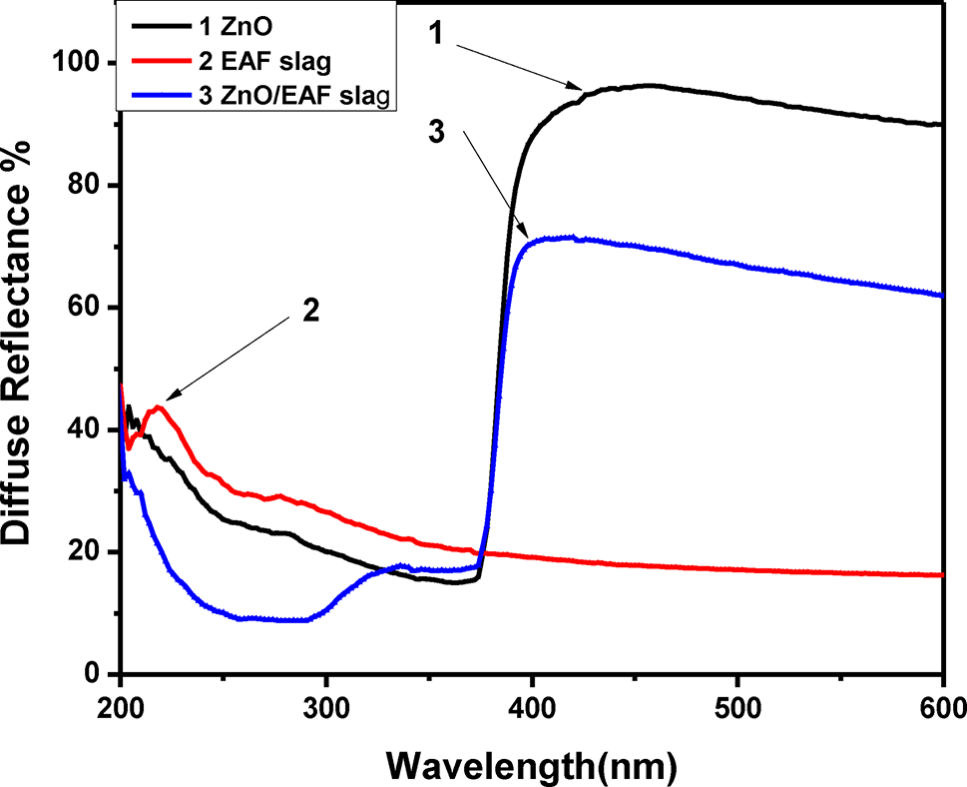
Diffuse reflectance spectra of for ZnO, EAF slag and ZnO doped EAF slag nanoparticles.

The diffuse reflectance spectrum (DRS) of homogenized powder samples were recorded by collecting scattered light from powdered samples using spectrophotometer in the wavelength range of (500–1000 nm)^30^. Figure 11 clears DRS for ZnO, EAF slag and ZnO doped EAF slag, the absorption maximum of the ZnO NPs is 420 nm, while it is 217 nm for EAF slag and 390 nm for ZnO doped EAF slag. Furthermore, the red shift can be related to sample agglomerations^31,32^, while the blue shift is related to nanoparticles reducing crystal size^33^. The energy band gap was calculated using Kubelka–Munk function:

2 $${\rm F(R)}=\frac{(1-{\rm R})^{2}}{2{\rm R}}$$

**Fig. 12:**
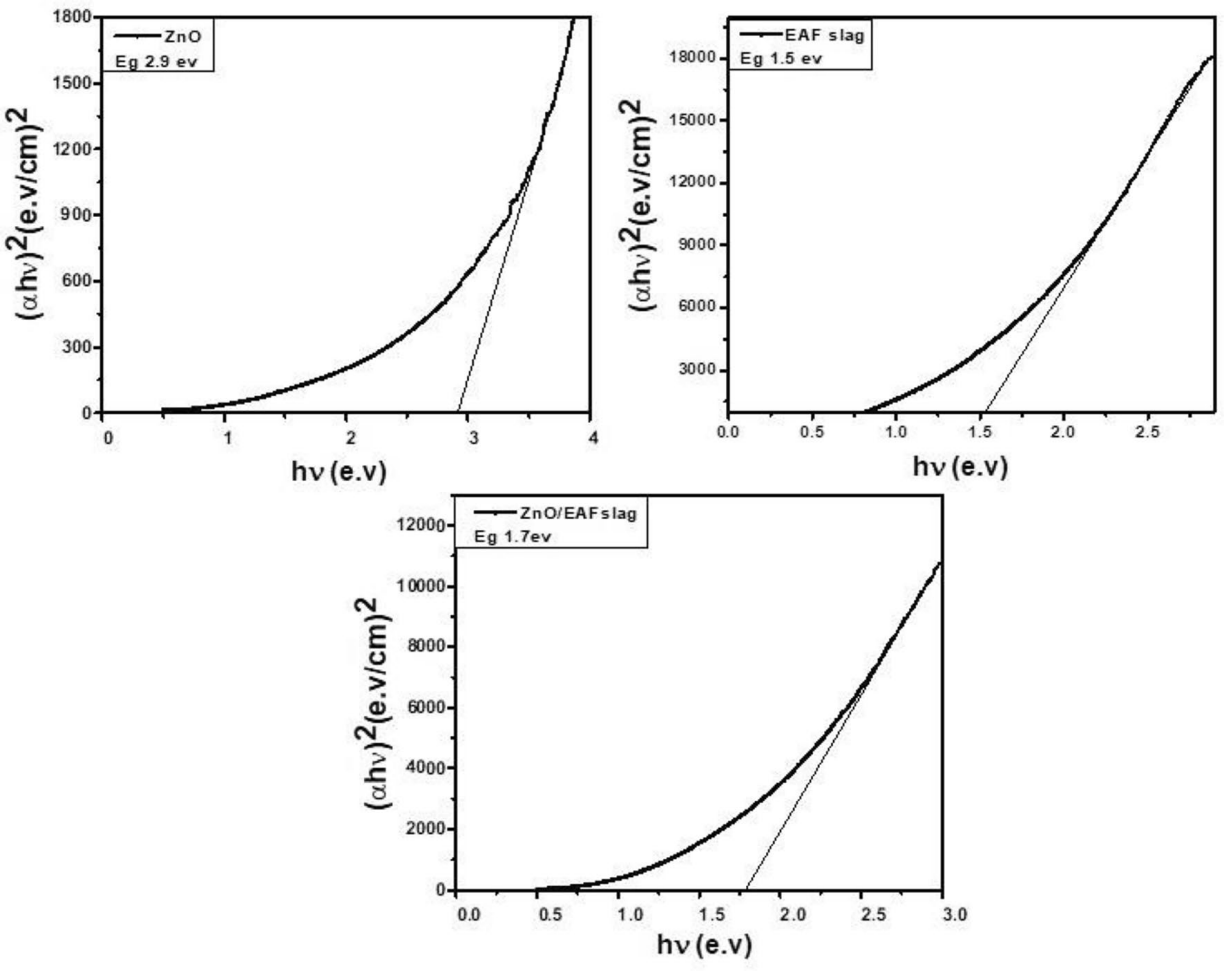
The relation between (αhν)^2^ and hν for ZnO, EAF slag and ZnO doped EAF slag nanoparticles.

Where (R) is the absolute value of reflectance and F(R) is equivalent to the absorption coefficient. The direct band gap of ZnO was estimated by plotting the relation of (αhν) ^2^ vs. E (hν) were used to determine E_g_ for the Photocatalytic made of ZnO, EAF slag and ZnO doped EAF slag NPs, as shown in Fig. 12. The obtained band gap energy (E_g_) values were 2.9, 1.5 and 1.7 eV for pure ZnO, EAF slag and ZnO doped EAF slag respectively. A reduction in the E_g_ refers to the change that happens when the concentration of ZnO nanoparticle doping of EAF slag increases. The decrease of E_g_ value can also be explained by the presence of Fe atoms content after doping with ZnO which can inhibit crystal growth. Moreover, the addition of Fe content doping to ZnO results in defects in ZnO crystals that lead to significant light absorption, which is crucial to raising ZnO’s photoactivity for visible light^34,35^.

### Photo luminescence

**Fig. 13:**
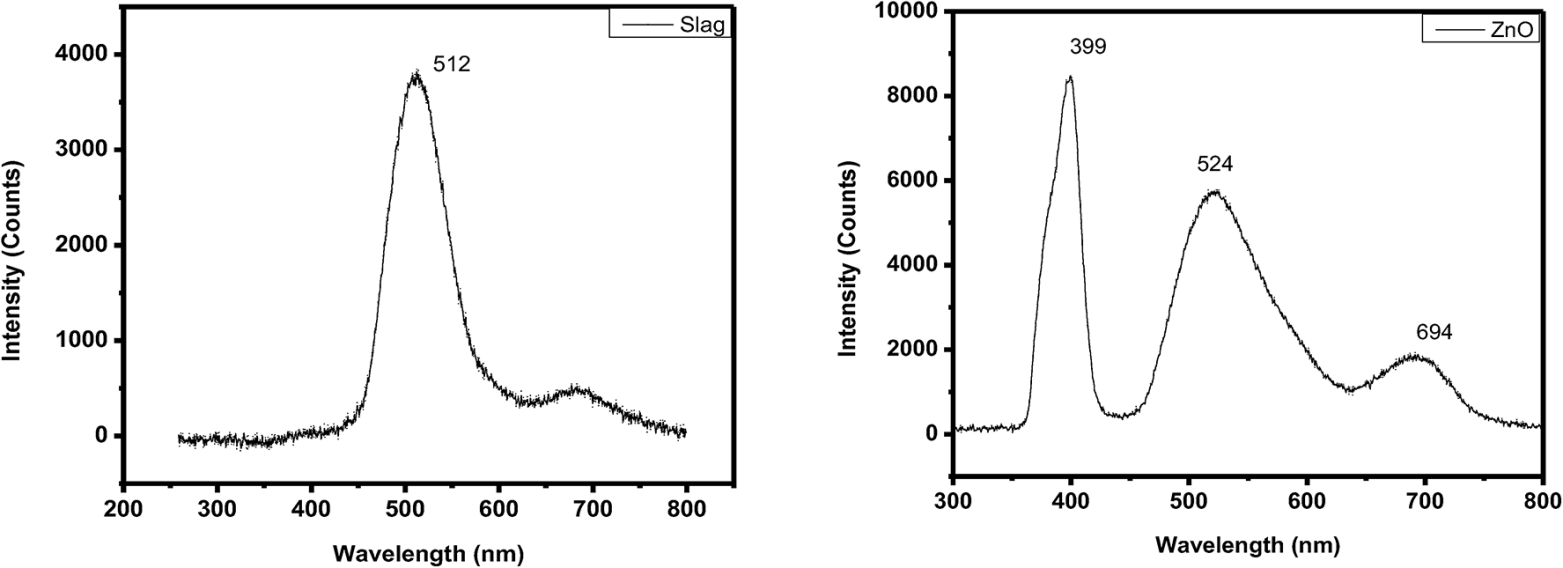
Photoluminescence spectra of iron slag & ZnO.

Room temperature photoluminescence spectroscopy (PL) was used for optical measurements, making it possible to investigate the electrical structure of semiconductor materials and structures effectively. PL spectrometer in Fig. 13 consists of He-Cd laser (532 nm), Sumitomo optical He closed cycle cryostat coupled with THR 1000 mono-chromator (Jobin-Yvon), excitation power source 20mW, and FTIR spectrometer Nicolet 5700. The following spectral range can be measured with great precision and sensitivity using the PL setup: 100–800 nm.

In the ultraviolet (UV) spectrum, the samples show a strong sharp narrow peak at around 399 nm. This is the near band edge (NBE) emission that results from the recombination of free excitons in ZnO^36^. The amount of free excitons participating in radiative annihilation is responsible for the fluctuation in peak intensity of UV emission. Furthermore, in the visible range, ZnO shows a low-intensity peaks at ~ 694 nm in addition to a wide peak centered at around 524 nm^36^.

While for iron slag it exhibits a prominent band of luminescence at 512 nm, which is caused by exciton emission and visible even at ambient temperature. The iron slag exhibits a low exciton state as a result of excitation between the Fe (4s) states-derived Conduction Band (CB) and the mixed Fe (3d) and O (2p) states that make up the Valence Band (VB)^37^.

### The mechanism for photocatalytic degradation of MR dye

When light photon interacts with the slag surface, an electron from the valence band (vb) moves to the conduction band (cb), creates a positively charged hole in the valence band (hvb+). Meanwhile, the conduction band (ecb−) experiences an increase in negative charge, leading to the formation of photocatalytic active centers on the surface of EAF slag NPs. In the case of both slag and ZnO, it was discovered that the band associated with MR absorption reduced over time. The photocatalytic degradation of MR by EAF slag NPs was found to be enhanced in the presence of Zn + ions. Moreover, SiO_2_ NPs was found to be photo-excited via sun light and showed an absorption band at around 413 nm, which is attributed to the charge transfer from bonding orbital of slag to 2p non-bonding orbital of non-bridging oxygen in ZnO.

## Conclusion

The use of EAF slag when mixed with ZnO may be a novelty in this aim of enhancing the removing of Methyl Red dye (MR) from industrial effluent by using inorganic materials such as ZnO doping with an activator such as EAF slag. This is environmentally beneficial for both targets of utilizing scrap waste from one side and removing dyes, purifying recycled water and make it possible to reuse it from the other side. XRD sharp and strong peaks of EAF slag and ZnO NPs indicate to the high crystallinity for the samples. in case of dosage 1gm ZnO, the increasing efficiency reached up to 85% in the first hour and after that decrease due to interaction of ZnO with MR dye solution. After 20% EAF slag addition the efficiency of MR dye removal improved and increased to 50% in the first hour and reached to 75% in the second hour. Then we noticed that when EAF slag was 25% the maximum efficiency we get was 10% removal only and these maybe due to the interaction between EAF slag and MR dye molecules. In summary, after two hours of testing, ZnO nanoparticles soaked with 20% EAF slag waste were shown to be the most effective in eliminating methyl red (MR), attaining ~ 96% elimination and a totally transparent solution.

## Data Availability

The corresponding author can provide the datasets created and/or analyzed during the current work upon justifiable request.D. A. Wissa, Nadia F. Youssef, Christen Tharwat.
